# Calcinosis Cutis in a Patient With Systemic Lupus Erythematosus: A Case Report

**DOI:** 10.7759/cureus.98457

**Published:** 2025-12-04

**Authors:** Mohammed Osman Ahmed Osman, Ziryab Imad, Yassin Abdalla, Salih Boushra Hamza, Mohammedelmuntaga Gafar, Obada Mohamed Ahmed Ali

**Affiliations:** 1 Internal Medicine, The National Ribat University, Khartoum, SDN; 2 Internal Medicine and Rheumatology, University of Bahri, Khartoum, SDN; 3 Internal Medicine, Omdurman Islamic University Faculty of Medicine and Health Sciences, Khartoum, SDN; 4 Internal Medicine, Omdurman Islamic University, Omdurman, SDN; 5 Internal Medicine and Rheumatology, Alzaiem Alazhari University Faculty of Medicine, Khartoum, SDN; 6 Internal Medicine, University of Bahri College of Medicine, Khartoum, SDN

**Keywords:** calcification, calcinosis cutis, connective tissue disorder, dystrophic, systemic lupus erythematosus

## Abstract

Systemic lupus erythematosus (SLE) is a systemic, remitting, and relapsing autoimmune disease. It is associated with deficiency of early complement proteins, impaired clearance of immune complexes, and numerous clinical manifestations and morbidities.

We report the case of a 40-year-old woman with a known diagnosis of systemic lupus erythematosus (SLE) who presented to the outpatient department with painful skin lesions over the left and right popliteal regions. She also reported generalized body aches, persistent fever that worsened at night, pain in multiple small and large joints, pruritus, hair loss, dry mouth, and right hip swelling for five years. Blood investigations revealed a positive antinuclear antibody (ANA) titer of 1:160 and positive anti-dsDNA antibodies, with normal serum calcium and phosphorus levels and an elevated erythrocyte sedimentation rate (ESR). Radiographs of both knees and the pelvis demonstrated multiple soft tissue calcifications of varying sizes. Histopathological examination revealed dense, massive calcified material in the subcutaneous tissue, confirming a diagnosis of calcinosis cutis. Surgical excision of the lesion was performed, and immunosuppressive agents were administered. However, one month later, the patient re-presented with a persistent infected wound that required antibiotic therapy. She subsequently received further medical treatment and underwent repeat surgical excision.

Calcinosis cutis is an exceedingly rare but potentially unfavorable complication of systemic lupus erythematosus, with only a limited number of cases reported in the literature, and it is most often associated with underlying cutaneous pathology. Clinicians must maintain a high index of suspicion to assure early diagnosis and treatment.

## Introduction

Calcinosis cutis is a rare, chronic, and potentially disabling condition characterized by the deposition of insoluble calcium salts in the skin, subcutaneous tissue, joints, ligaments, and muscles. It may result in significant pain and functional impairment and is most commonly associated with autoimmune connective tissue diseases [1]. Among these, systemic sclerosis and dermatomyositis show the strongest association with its development [2]. Only a limited number of cases have been reported in patients with systemic lupus erythematosus (SLE). Soft tissue calcifications are classified into five major types: dystrophic, metastatic, idiopathic, tumoral, and calciphylaxis [3-10]. The dystrophic type occurs in areas of tissue destruction with normal serum calcium and phosphate levels, whereas the metastatic type develops in the setting of hypercalcemia and/or hyperphosphatemia. Calcinosis cutis usually progresses gradually and is often asymptomatic [5].

Calcinosis cutis is most commonly associated with underlying skin pathology. This study reports a rare coexistence of calcinosis cutis and systemic lupus erythematosus. Further research is needed to better elucidate the underlying mechanisms of this association.

## Case presentation

A 40-year-old Sudanese woman presented to the outpatient department (OPD) with generalized body aches, persistent nocturnal fever, polyarticular pain involving the distal interphalangeal joints, bilateral wrists, shoulders, knees, and ankles, along with pruritus, hair loss, and dry mouth with gingival ulceration but no ocular dryness. She also reported a five-year history of right hip swelling. Three years ago, the patient developed a small, rounded, painless skin swelling in the left popliteal area. It lasted only for 10 days, then disappeared spontaneously. One year later, the skin lesion recurred at the same site and also appeared in the right popliteal area, presenting as a hard, elongated, darkly pigmented elevation that was painful on palpation. She had been diagnosed with systemic lupus erythematosus (SLE) five years earlier but was not receiving long-term therapy. Her medical history was notable for epistaxis secondary to immune thrombocytopenic purpura (ITP) and a prior intensive care unit admission eight years earlier due to severe pancytopenia.

**Figure 1:**
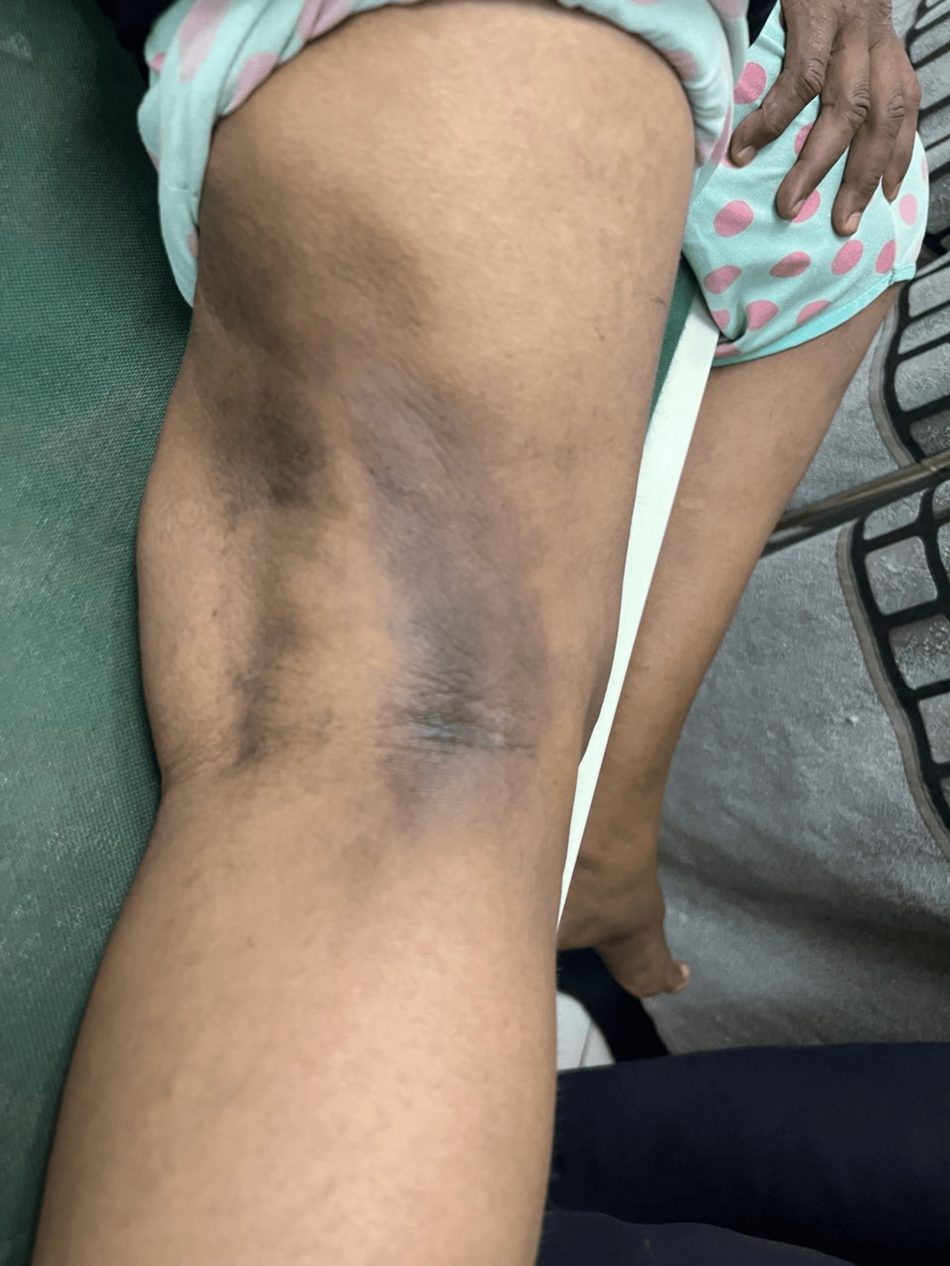
Skin pigmentations over the popliteal area

On examination, the patient appeared pale but was alert and hemodynamically stable. There was tenderness in both the right and left iliac fossae, with surgical scars noted over the left iliac fossa and the right shoulder. A skin rash was observed in the axillae and on the left side of the neck, along with hyperpigmentation over the right popliteal region (Figure 1). 

Blood investigations revealed an unremarkable full blood count (FBC) and an elevated erythrocyte sedimentation rate (ESR). Serum calcium and phosphate were normal. Liver enzymes and serum albumin were normal. Complement levels (C3 and C4) were within normal limits (Table 1).

**Table 1:** Laboratory findings ESR: Erythrocyte sedimentation rate; CRP: C-reactive protein; RBCs: Red blood cells; MCV: Mean corpuscular volume; MCH: Mean corpuscular haemoglobin; MCHC: Mean corpuscular haemoglobin concentration; RDW: Red cell distribution width; TWBCs: Total white blood cells; ALT: Alanine transaminase; AST: Aspartate transaminase; ALP: Alkaline phosphatase, GGT: Gamma-glutamyl transferase.

Test	Result	Normal Range
TWBC	3.6 cells/mm^3^	4-11 × 10^3^/µL
RBC	5.1 million/mm^3^	4.0-5.2 million/mm^3^
HGB	12.8 g/dL	12.0-16.0 g/dL
HCT	37.20%	37-47%
MCV	72.4 fL	76-96 fL
MCH	24.8 pg/cell	27-32 pg/dL
MCHC	34.40%	33-37%
PLT	256,000/mm^3^	150,000-400,000/mm^3^
LYM (%)	52%	20-45%
MXD (%)	3%	2-10%
NEUT (%)	43%	40-70%
Basophils (%)	0.20%	0-1%
Eosinophils (%)	0.20%	2-6%
RDW-CV	18.60%	10-15%
CRP	4.0 mg/L	Negative: less than 10 mg/L
ESR	50 mm/h	Up to 10 mm/h
Renal function tests		
Creatinine	0.7 mg/dL	0.6-1.1 mg/dL
Blood urea	30 mg/dL	15-50 mg/dL
Sodium	140 mmol/L	135-150 mmol/L
Potassium	3.5 mmol/L	3.6-5.5 mmol/L
Serum calcium	2.5 mmol/L	2.1-2.8 mmol/L
Serum phosphorous	1.7 mmol/L	1.0-1.5 mmol/L
Liver function tests		
ALT	11 U/L	10-60 U/L
AST	30 U/L	7-40 U/L
ALP	56 U/L	30-85 U/L
GGT	15 U/L	5-55 U/L
Total protein	7.7 g/dL	6.4-8.2 g/dL
Albumin	3.9 g/dL	3.4-5 g/dL
Globulin	3.8 g/dL	2.9-3.7 g/dL
Total bilirubin	0.2 mg/dL	Up to 1.5 mg/dL
Direct bilirubin	0.1 mg/dL	0.05-0.3 mg/dL
Indirect bilirubin	0.1 mg/dL	Less than 0.7 mg/dL

Antinuclear antibody (ANA) testing was positive with a nucleolar and fine cytoplasmic granular pattern at a titer of 1:160, along with a positive anti-double-stranded DNA (anti-dsDNA) antibody. All other ANA subtypes, including Scl-70, SS-A, and SS-B, were negative. Rheumatoid factor and anti-cyclic citrullinated peptide (anti-CCP) antibodies were also negative (Table 2).

**Table 2:** ANA profile test ANA: Antinuclear antibody; anti-dsDNA: Anti-double-stranded DNA; anti-CCP: Anti-cyclic citrullinated peptide antibody; anti-RNP: Anti-ribonucleoprotein antibody; AMA: Anti-mitochondrial antibody; anti-Scl-70: Anti-scleroderma-70 antibodies; SmD1: Smith antibodies; anti-SS–A/Ro 60 KD: Sjogren anti-SS-A; anti-SS–A/Ro 52 KD: Sjogren anti-SS-B; anti-U1-snRNP: U1 small nuclear ribonucleoprotein particle.

ANA profile		
ANA global titre	1/160 (homogenous pattern)	>1/80
ANA screening	Positive	Negative: <12.0, doubtful: 12-18, positive: >18.0
Anti-dsDNA	Positive	Negative
Anti-SS-A/Ro 60 KD	Negative	Negative
Anti-SS-A/Ro 52 KD	Negative	Negative
Anti-Jo-1	Negative	Negative
Anti-nucleosome	Negative	Negative
Anti-histone	Negative	Negative
Anti-SmD1	Negative	Negative
Anti-PCNA	Negative	Negative
Anti-PO	Negative	Negative
Anti-Scl-70	Negative	Negative
Anti-CENPB	Negative	Negative
Anti-AMA-M2	Negative	Negative
Anti-U1-snRNP	Negative	Negative
Anti-SSB/La	Negative	Negative
Anti-PM/Scl	Negative	Negative
Anti-Mi-2	Negative	Negative
Anti-Ku	Negative	Negative
Rheumatoid factor	Negative	Negative: <12.0, doubtful: 12-18, positive: >18.0
Anti-CCP	Negative	Negative: <12.0, doubtful: 12-18, positive: >18.0

**Figure 2:**
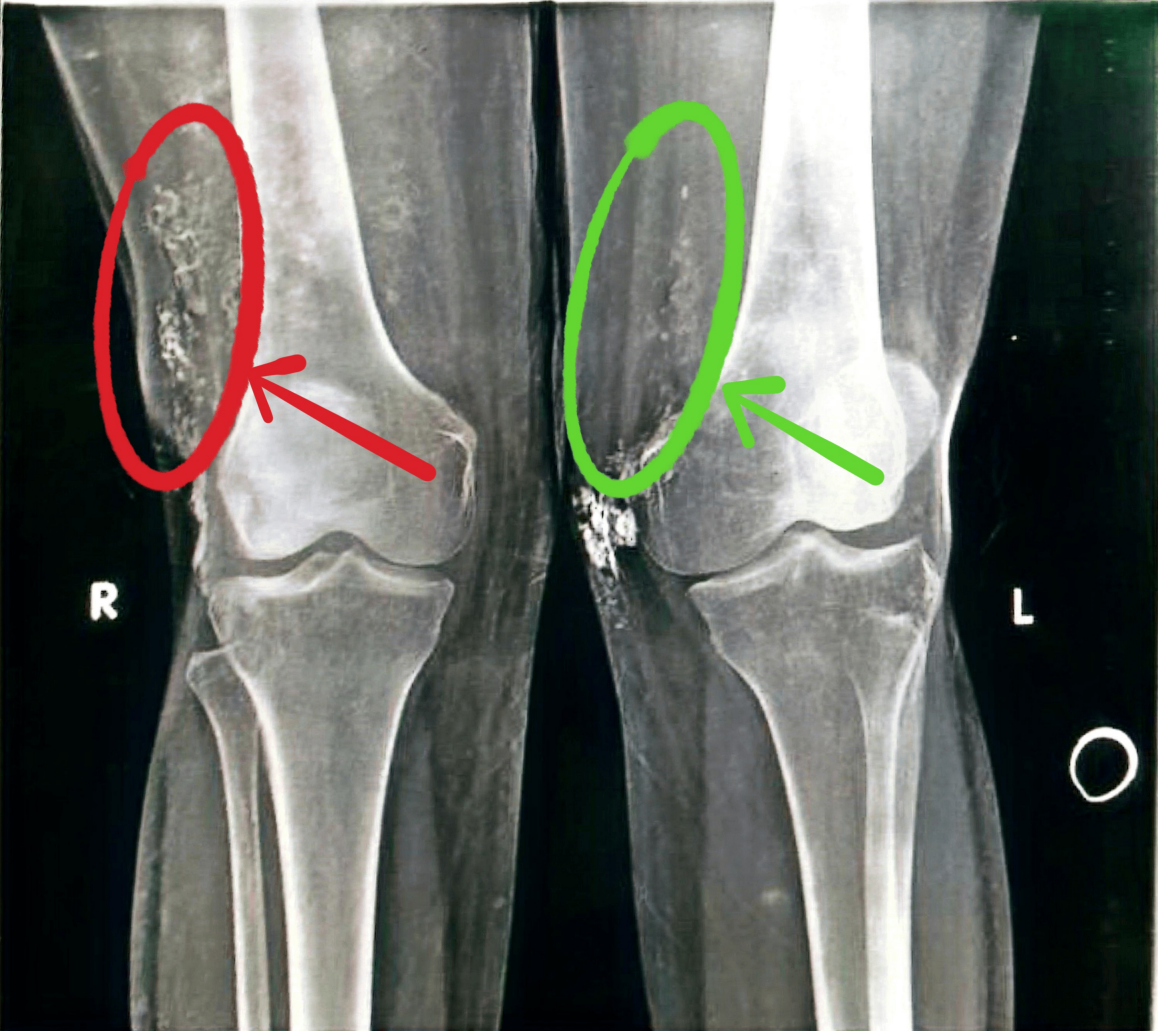
Soft-tissue calcifications Frontal radiograph of both knee joints showing multiple soft-tissue calcifications on both sides of the distal thigh, more on the right side. Red circle and arrow: soft-tissue calcifications on the right thigh; green circle and arrow: soft-tissue calcifications on the left thigh

Viral screening was negative for HBV, HCV, and HIV (Table 3). Both, knees and pelvis showed multiple soft-tissue calcifications of various sizes on X-rays (Figure 2).

**Table 3:** Viral screening: HIV, HPV, HCV HIV: Human immunodeficiency virus; HPV: Human papillomavirus; HCV: Hepatitis C virus.

Viral screening		
HIV	Negative	Negative
HBV	Negative	Negative
HCV	Negative	Negative

Subsequently, an excisional biopsy was performed, and oral prednisolone at a dose of 20 mg was initiated and later tapered following the procedure. Two skin biopsy specimens measuring a combined size of 1 × 0.5 × 1 cm were obtained. Histopathological examination revealed irregular, intensely basophilic, well-circumscribed calcified deposits within the dermis and subcutaneous tissue, surrounded by inflammatory infiltrates and giant cell reactions. These findings were consistent with a diagnosis of calcinosis cutis.

**Figure 3:**
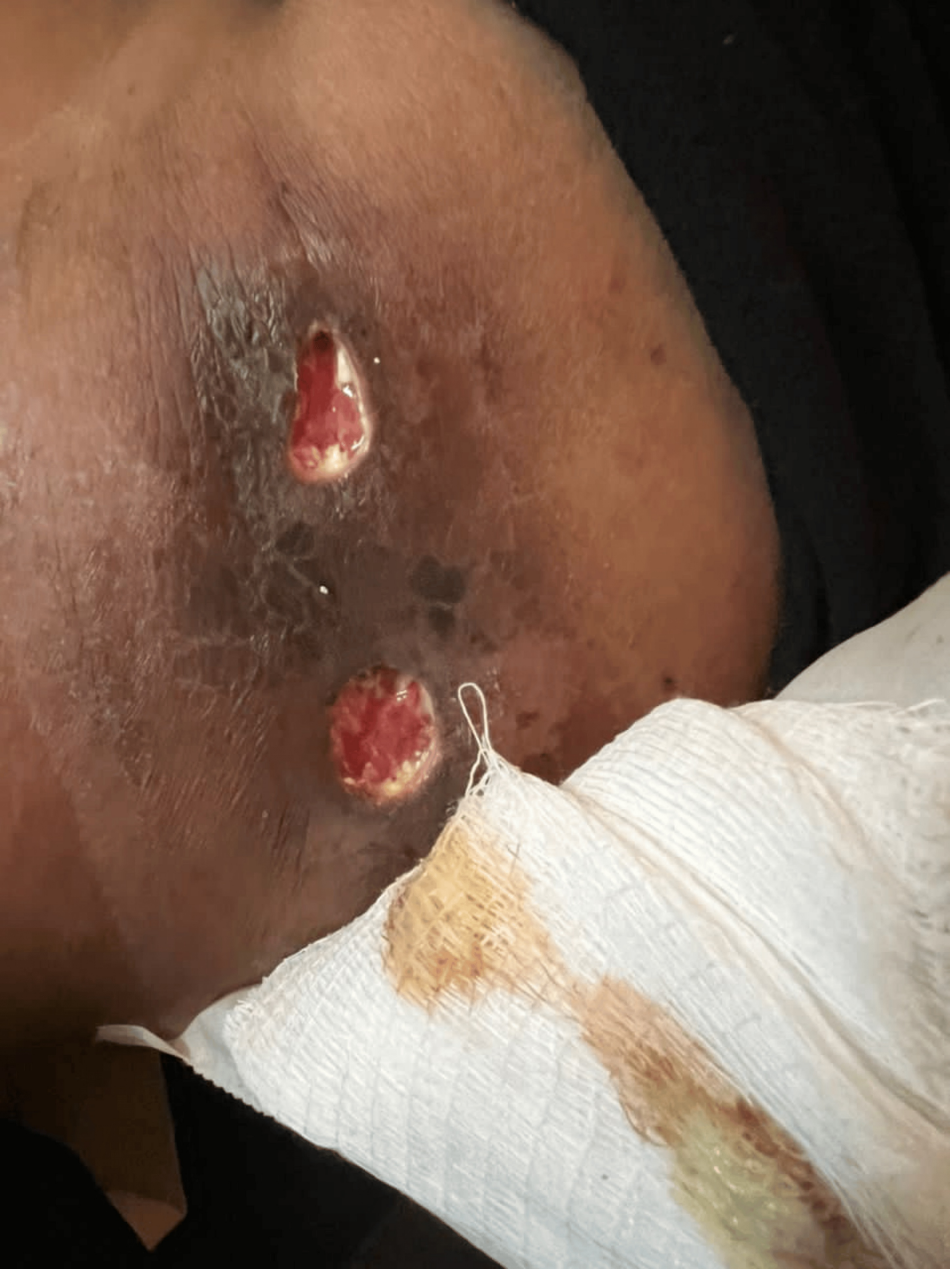
Infected wound one month after treatment

Consequently, she received hydroxychloroquine 200 mg twice daily, mycophenolate mofetil 500 mg once daily for three days, calcium supplement 500 mg once daily, and three Omega-3 tablets per day. One month later, she returned to the OPD with an infected wound (figure 3). Treatment with oral amoxicillin and clavulanic acid (1 g tablets twice per day) was prescribed, and she was then shifted to mycophenolate 360 mg twice daily. The subcutaneous lesions remained unchanged. 

## Discussion

Calcium deposition within the dermis, epidermis, and subcutaneous tissue is observed in a variety of rheumatological diseases [11-12]. In our patient, multiple skin lesions developed five years after the diagnosis of systemic lupus erythematosus, predominantly involving the popliteal and gluteal regions. Calcinosis cutis is classified into five subtypes. The idiopathic type refers to calcium deposition in normal tissues with normal serum calcium and phosphate levels [13-15]. The dystrophic type is characterized by aberrant calcium deposition in the skin, subcutaneous tissue, muscles, or tendons with sparing of the viscera [15-16]. This subtype is rarely associated with SLE but is more frequently observed in scleroderma and dermatomyositis. In the metastatic type, calcium salt deposition is observed in normal tissues in the presence of elevated serum calcium and phosphate levels [13-15]. The tumoral type is associated with rapid progression to skin ulceration and necrosis. Calciphylaxis typically occurs in patients with chronic kidney disease undergoing dialysis [13,14]. Based on these classifications, our case is most consistent with the dystrophic type. 

The disease primarily affects the buttocks and extremities and manifests as small cutaneous nodules, although the pathophysiology of calcinosis cutis remains unclear. Several mechanisms have been proposed to explain the process of calcification, with local inflammation widely believed to play a central role in its development [14]. One study suggests that extracellular pyrophosphates, which normally inhibit calcium deposition, may be hydrolyzed by damaged lysosomes, leading to the release of alkaline phosphatase and subsequent calcification [17]. Another hypothesis proposes that calcification represents a by-product of local ischemia resulting from steroid-induced hypertrophy of fat cells under pressure [18].

Several studies have documented the benefits of surgical management for calcinosis cutis [13,19,20]. Surgical intervention is indicated in the presence of painful masses, recurrent infection, ulceration, local functional impairment, or for cosmetic reasons. However, recurrence after surgery has been reported [21]. Other studies have shown that diltiazem, a calcium channel blocker, may occasionally result in improvement of calcification [22,23]. In our case, the patient underwent surgical excision of the lesions. In summary, we report calcinosis cutis as a complication in a patient with SLE. Two previously reported cases demonstrated limited therapeutic benefit; the first showed a poor response after three months of diltiazem therapy, while the second did not undergo any intervention because the patient was asymptomatic [6,8].

Both calcinosis circumscripta and calcinosis universalis have been reported in association with SLE, with the circumscripta type being more common [24]. The calcinosis universalis observed in our case is typically associated with long-standing SLE and shows a female predilection, consistent with our patient’s five-year history of poorly controlled disease. Most previously reported cases involved females in their fourth decade of life [8,24-26].

Calciphylaxis is an important differential diagnosis in the context of SLE, as it has been reported in association with lupus nephritis, with or without end-stage renal disease; the former is referred to as non-uremic calciphylaxis. Although calciphylaxis primarily affects blood vessels, dermatological manifestations such as purpuric patches and plaques have been described. Differentiation between calciphylaxis and calcinosis cutis is critical, as calciphylaxis requires specific treatment with sodium thiosulfate [27].

## Conclusions

Calcinosis cutis is a disorder characterized by pathologic calcium deposition in the cutaneous and subcutaneous layers of the skin. It is commonly associated with systemic sclerosis and dermatomyositis and represents a rare extra-articular manifestation of systemic lupus erythematosus (SLE). Although the association between calcinosis cutis and SLE has been reported in the literature, it remains uncommon. A high index of suspicion is necessary to ensure early diagnosis and treatment, and further studies are required to elucidate the underlying mechanisms and to determine optimal therapeutic strategies for patients with SLE.
